# Clear cell carcinoma arising from abdominal wall endometriosis: a unique case with bladder and lymph node metastasis

**DOI:** 10.1186/1477-7819-12-51

**Published:** 2014-03-05

**Authors:** Haiyuan Liu, Jinghua Leng, Jinghe Lang, Quancai Cui

**Affiliations:** 1 Department of Obstetrics and Gynecology, Peking Union Medical College Hospital, Peking Union Medical College, Chinese Academy of Medical Science, Beijing, China; 2 Department of Pathology, Peking Union Medical College Hospital, Peking Union Medical College, Chinese Academy of Medical Science, Beijing, China

## Abstract

The malignant transformation of abdominal wall endometriosis is a rare event and poorly understood. Less than 30 cases have been reported in the literature. Most of the reported cases have a solitary tumor in the abdominal scar. A few cases have metastasis. Here we report a case of clear cell carcinoma in abdominal wall endometriosis with bladder and lymph system metastasis. The patient had a history of abdominal wall endometriosis and recently developed symptoms of urgent urination and inguinal mass. Physical examination and a computed tomography (CT) scan detected lymph node metastasis. CT and cystoscopy confirmed bladder involvement. The patient underwent extensive surgery and chemotherapy. Pathological analysis made a diagnosis of clear cell carcinoma with bladder and lymph node metastasis. The patient was followed up and died of the disease. Symptoms of bladder invasion and lymph node spread could be a sign of malignant transformation. Local invasion and lymph node spread are two important forms of tumor metastasis. Extensive lymph nodes metastasis might be related with poor prognosis.

## Background

Endometriosis is defined as the presence of endometrial glands and stroma outside the uterine cavity. It affects about 15% of women of childbearing age. The most common site of endometriosis is in the pelvic cavity but it occasionally occurs in the extrapelvic area. Abdominal wall endometriosis represents 1% to 2% of all endometriosis lesions [1]. It is mostly found in umbilicus and incisional scars, especially following caesarean sections (CSs). Malignant transformation of abdominal wall endometriosis is a rare event and poorly understood. Most cases in the literature had local invasion. Here we report a unique case of clear cell carcinoma in abdominal wall endometriosis with bladder and lymph system metastasis.

## Case presentation

**Figure 1:**
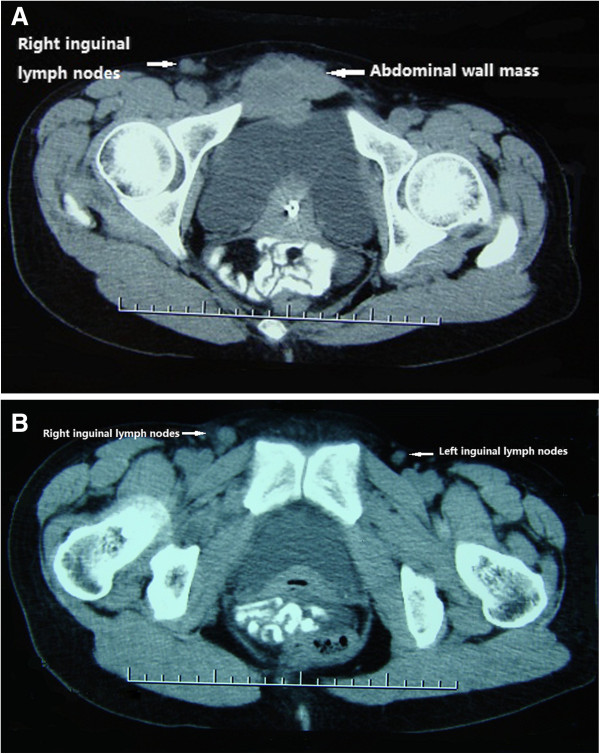
**Computed tomography scans.** The abdominal wall mass had invaded the bladder **(A)** and the inguinal lymph nodes **(A, B)**.

**Figure 2:**
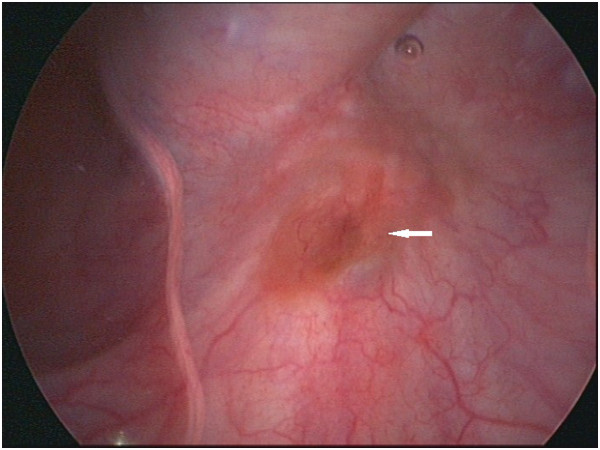
Cystoscopy image showing the abdominal wall mass had penetrated the roof of the bladder near the membrane (arrow).

**Figure 3:**
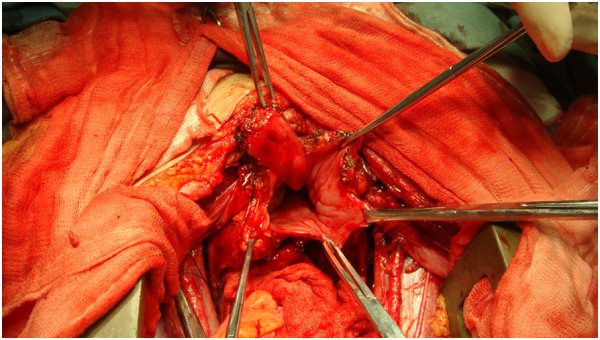
Photograph taken during laparotomy showing the abdominal mass had invaded the roof of the bladder.

**Figure 4:**
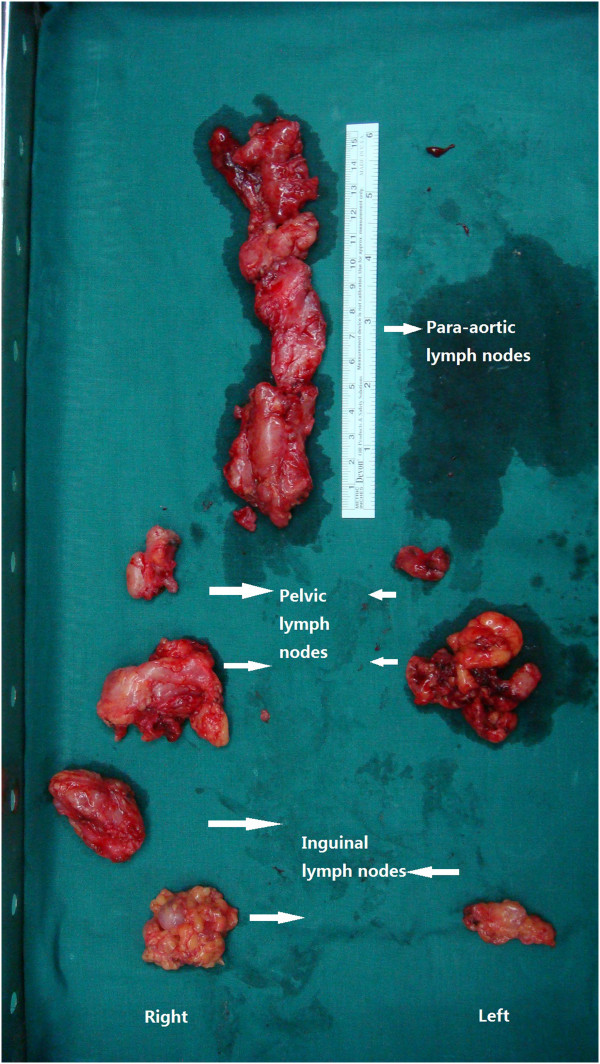
**Samples from lymphonectomy.** These showed that the inguinal lymph nodes, pelvic lymph nodes and the para-aortic lymph nodes were all enlarged.

**Figure 5:**
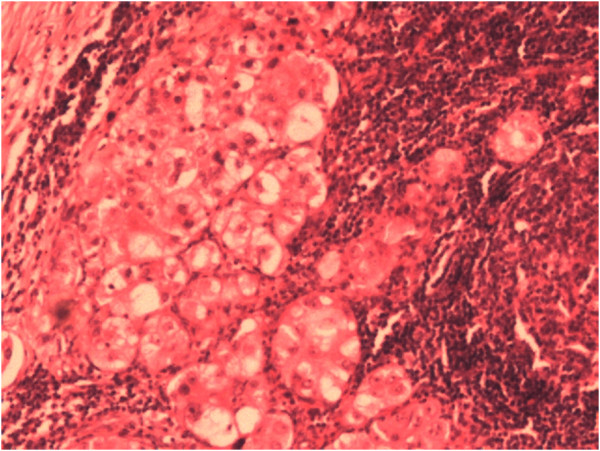
Pathological finding of clear cell carcinoma within the lymph node.

A 39-year-old woman with a history of abdominal wall endometriosis presented to our clinic complaining of urgent urination and an emerging inguinal mass. She had a CS due to a breech presentation in 1994. She noticed a mass in the CS scar with cyclic pain in 1999. The mass was excised and diagnosed as abdominal scar endometriosis histologically. The margin was free and no further treatment was given after surgery. In 2004 a similar mass with cyclic pain was detected in the scar again. The mass grew gradually and was diagnosed as recurrent abdominal wall endometriosis without further treatment. She also developed urgent urination and dysuria during her menstrual period. In the last 4 months she noticed masses emerging in the bilateral inguinal area with mild pain.A physical examination showed a solid 6 cm × 5 cm mass without a clear borderline near the symphysis pubic in the previous longitudinal scar. Two enlarged lymph nodes with diameters of 2 cm and 1 cm in the right groin, and one lymph node with a diameter of 1 cm in the left groin were detected. Serum CA125 was 22.1 U/ml. Computed tomography (CT) scans showed that the mass had invaded the bladder and that the inguinal lymph nodes were enlarged (Figure 1). Cystoscopy showed that the mass had penetrated the roof of the bladder near the membrane (Figure 2). Malignant transformation of abdominal wall endometriosis was highly suspected before surgery.During laparotomy, it was seen that the mass had invaded the whole abdominal wall to the peritoneum and the roof of the bladder was also involved (Figure 3). There was no endometriosis detected in the pelvic cavity. A frozen section pathological analysis was done and malignant disease was confirmed. Therefore, the woman underwent extensive surgery including: partial bladder excision, hysterectomy, bilateral adnexectomy, omentectomy and lymph node excision. The inguinal lymph nodes, pelvic lymph nodes and para-aortic lymph nodes were all enlarged (Figure 4).Pathological examination gave a diagnosis of clear cell carcinoma in a background of endometriosis with metastasis to the bladder and the lymph nodes (Figure 5). There were 8/8 positive bilateral inguinal lymph nodes, 18/21 positive pelvic lymph nodes and 6/6 positive para-aortic lymph nodes.

The patient recovered uneventfully but refused further treatment after three cycles of chemotherapy with carboplatin and paclitaxel. She sought traditional Chinese herbal medication. Ten months after chemotherapy, tumor recurrence was detected in the pelvic cavity and the patient died of the disease two months later.

### Discussion

Malignant transformation of endometriosis is quite rare, affecting 1% of women suffering from endometriosis. The most common sites of malignant transformation of endometriosis are the ovaries. About 20% of cases occur in extragonadal sites including the rectovaginal septum, colon and vagina [2]. The malignant transformation of endometriosis in the abdominal wall is a rare event and less than 30 cases have been reported in the literature.

Sampson [3] proposed three criteria to diagnose a malignant transformation of the endometriosis: demonstration of both neoplastic and benign endometrial tissue in the tumor, histological type of tumor compatible with endometrial origin and no other primary site identified. Scott [4] added a fourth criterion of transition between histological benign endometriosis and carcinoma, which was defined as atypical endometriosis with cytological or architectural atypia in the endometriotic glands [5]. These criteria were based on the characteristics of malignant transformation of ovary endometriosis. Only a few cases of malignant abdominal wall endometriosis have fulfilled all four criteria [6]. All cases of malignant transformation of abdominal wall endometriosis in the literature had a history of surgery that caused the iatrogenic dissemination of the endometrium and a histological type of tumor compatible with an endometrial origin and no other primary site was identified [2,7-9].

Malignant transformation of endometriosis in the abdominal wall can invade all layers of the abdominal wall and grow as large as 10 cm in diameter [6]. However, there is no report of bladder invasion in the literature. In the present case, the mass arose near the symphysis pubis just above the roof of the bladder. When the neoplasm grew and invaded the wall of the bladder, the patient developed bladder-stimulating symptoms. CT scans clearly showed the bladder invasion since the margin between the mass and the wall of the bladder was visible. Cystoscopy confirmed bladder metastasis because the involvement of the bladder wall and mucosa can be identified clearly.

Local invasion is one important biological characteristics for malignant transformation of endometriosis in the abdominal wall; however, it can also spread through the lymph system. Three other cases with lymph metastasis have been reported in the literature (Table 1). For the four cases, the average latency was 16.75 years. The carcinomas had a wide range of local invasion to all layers of the abdominal wall with a diameter of at least 5 cm. Three of the four cases had a histological type of clear cell carcinoma and one had mixed endometrioid and serous carcinoma. Pre-surgical evaluation and diagnosis of lymph node dissemination is difficult. Lymph metastasis was missed in both preoperative magnetic resonance imaging (MRI), surgical exploration and postoperative CT in cases 1 and 3. During the repair of the surgical wound, a 5-cm lymph node was found near the right external iliac artery in case 1. In case 3, postoperative positron emission tomography with computed tomography (PET-CT) using 18-fluorodeoxyglucose showed the involvement of two left iliac nodes. In case 2 and our case, a bilateral inguinal mass with metastasis was detected before surgery and confirmed by an imaging scan. Only in our case, was a systemic lymph dissection conducted. The lymph invasion clearly demonstrated that the path for the lymph metastasis in the malignant transformation of endometriosis in the abdominal wall was from the inguinal lymph nodes to the para-aortic lymph nodes through the pelvic lymph nodes. This pathway is consistent with the route of lymph fluid reflux in the lower abdominal wall. Symptoms of emerging inguinal mass together with local physical examination and imaging scan might be valuable for detecting primary lymph nodes metastasis before surgery.

**Table 1:** Four cases of malignant transformation of endometriosis in the abdominal wall with lymph metastasis

**Case (reference)**	**Age**	**Time to onset**	**Tumor size**	**Histology**	**Lymph metastasis**	**Treatment**	**Outcome**
		**(years)**	**(cm)**		**(involved/total)**		
1. [6]	38	13	10	CCC	Right iliac lymph node (1/28)	TAH + BSO	4 month NED
						Omentectomy	
						Radical resection	
						Lymph node dissection	
						Chemotherapy	
2. [7]	53	21	5	CCC	Inguinal nodes (17/17)	TAH + BS	11 month DOD
					Pelvic lymph nodes (10/14)	Omentectomy	
						Radical resection	
						Lymph node dissection	
3. [9]	48	16	6	Mixed endometrioid and serous carcinoma	Left iliac lymph nodes (2/not mentioned)	Radical resection	15 month NED
						Curettage	
						Lymph node dissection	
						Chemotherapy	
						LH + BSO	
This case	39	17	6	CCC	Inguinal nodes (8/8)	Radical resection	12 month DOD
					Pelvic lymph nodes (18/21)	TAH + BSO	
					Para-aortic lymph nodes (6/6)	Omentectomy	
						Lymph node dissection	
						Chemotherapy and Chinese herbal medicine	

BSO, bilateral salpingo-oophorectomy; CCC, clear cell carcinoma; DOD, died of disease; NED, no evidence of disease; TAH, transabdominal hysterectomy. LH, laparoscopic hysterectomy.

The diagnosis of malignant transformation of abdominal wall endometriosis is still a challenge for gynecologists. There are no characteristic symptoms and markers during malignant transformation. Imaging can detect endometrioma and its fast growth. Malignant transformation is only suspected when the tumor has a solid or mixed component. In the present case, the patient developed urgent urination and an inguinal mass, which could be a sign of tumor invasion. Therefore, malignant transformation and metastasis were highly suspected before surgery. Accordingly, we were able to make a detailed plan and prepare for extensive surgery.

The best treatment for malignant transformation of endometriosis in the abdominal wall is unknown. Radical surgery with a wide resection is believed to be the primary treatment [6]. Chemotherapy based on carboplatinum and radiotherapy have been proposed without any evidence of improved prognosis. In all four cases with lymph metastasis, radical therapy together with hysterectomy salpingo-oophorectomy and lymph node excision were the standard treatment. Chemotherapy was administered to cases 1 and 3. Our patient had chemotherapy and used traditional Chinese herbal medicine. Cases 1 and 3 had limited or isolated unilateral iliac lymph node metastasis (one lymph node in case 1 and two in case 3). These two patients had no sign of recurrence over the short time of the follow-up. While case 2 and our case had extensive lymph nodes metastasis, these two patients died of the disease within one year. Extensive lymph node involvement indicated the late stage of the disease with multiple metastases. This might also correlate with a poor prognosis.

## Conclusions

Malignant transformation of endometriosis in the abdominal wall is a rare complication and poorly understood. Symptoms of bladder invasion and lymph node spread could be a sign of malignant transformation. Local and lymph system invasion are two important routes for tumor metastasis. Extensive lymph node metastasis is probably related with poor prognosis.

## Consent

Written informed consent was obtained from the patient for publication of this case report.

## Abbreviations

CS: Cesarean section; CT: computed tomography.

## Competing interests

The authors declare that they have no competing interests.

## Authors’ contributions

HL collected clinical information, searched the relevant literature and wrote the draft. JL provided the case, followed up the patient and approved the manuscript. JL and HS participated in the surgery and revised the manuscript. QC performed the pathological analysis and provided the images in the manuscript. All authors approved the final manuscript.
